# Real-world adverse event analysis of Adagrasib: Insights from the FAERS database

**DOI:** 10.1097/MD.0000000000044755

**Published:** 2025-09-26

**Authors:** Sidi Jian, Shuang Liang, Xiaohan Ma, Sheng Chen, Xiaofei Zhang, Xuan Lan, Peiling Zuo, Encun Hou, Sijing Tu

**Affiliations:** a Graduate School, Guangxi University of Chinese Medicine, Nanning, Guangxi, China; b School of Public Health and Management, Guangxi University of Chinese Medicine, Nanning, Guangxi Zhuang Autonomous Region, China; c Department of Oncology, Ruikang Hospital, Guangxi University of Chinese Medicine, Nanning, Guangxi, China.

**Keywords:** Adagrasib, adverse event, cancer, FAERS, seizure

## Abstract

Adagrasib is a potent and orally available small-molecule inhibitor that irreversibly targets the KRAS G12C mutant isoform. This innovative therapeutic agent represents a significant advancement in the field of oncology, offering a targeted approach to treating cancers driven by this specific mutation. The precision and potency of Adagrasib make it a promising candidate for improving patient outcomes in KRAS G12C-mutated cancers. However, alongside its therapeutic potential, it is crucial to thoroughly understand and evaluate the safety profile of Adagrasib. The safety assessment includes a detailed examination of the adverse events (AEs) associated with its use. Comprehensive evaluation of these AEs is essential to ensure that the benefits of the drug outweigh any potential risks to patients. This retrospective pharmacovigilance study evaluated AEs associated with Adagrasib using data from the FDA adverse event reporting system database. We collected adverse drug reaction data for Adagrasib from the fourth quarter of 2022 to the first quarter of 2025. After standardizing the data, we applied several signal quantification methods, including the reporting odds ratio, proportional reporting ratio, Bayesian confidence propensity for neural networks, and Multi-item Gamma Poisson Shrinker (MGPS), for analysis. In this analysis of 556 adverse drug event reports where Adagrasib was identified as the primary suspect, we discovered 38 preferred terms across 20 system organ classifications. The 3 most prevalent system organ classifications were general disorders and administration site conditions, gastrointestinal disorders, and nervous system disorders. Notably, this study uncovered several new, previously unlabeled adverse reactions, including skin hyperpigmentation and seizures. These findings highlight the importance of ongoing pharmacovigilance to ensure comprehensive understanding of a drug’s safety profile. These findings highlight the need for continued monitoring and understanding of Adagrasib potential risks. Further research is necessary to confirm these associations and address any previously unrecognized safety concerns.

## 1. Introduction

Kirsten rat sarcoma viral oncogene homolog (KRAS) stands out as a membrane-associated guanosine triphosphatase, boasting the highest mutation frequency among all identified oncogenes across various human cancers.^[1,2]^ Specifically, non-small cell lung cancer cases demonstrate a prevalence of KRAS mutations, accounting for approximately 25% of incidences,^[3]^ with the KRASG12C point mutation in codon 12 occurring in about 14% of lung adenocarcinomas.^[4]^ Historically deemed undruggable, recent advances have changed this perception by targeting KRASG12C mutations.^[1,5]^ Adagrasib is a potent, orally available, small-molecule covalent inhibitor of KRASG12C, that irreversibly and selectively binds KRASG12C in its inactive, GDP-bound state.^[6]^ Adagrasib, a potent, orally available, small-molecule covalent inhibitor of KRASG12C, irreversibly and selectively binds KRASG12C in its inactive, GDP-bound state.^[7]^ Adagrasib possesses favorable characteristics, including a lengthy half-life (23 hours), dose-dependent pharmacokinetics, and penetration of the central nervous system.^[8]^ Clinical efficacy of Adagrasib has been observed in patients with KRASG12C-mutated solid tumors, encompassing non-small cell lung cancer, colorectal, pancreatic, and biliary tract cancers.^[9–11]^ These findings herald a significant breakthrough in the realm of oncology, marking a pivotal moment where what was once considered a formidable obstacle – KRAS mutations – now faces a targeted, efficacious intervention. The journey from the “undruggable” label to the tangible clinical efficacy of Adagrasib illuminates the power of innovative research and underscores the transformative potential of precision medicine in combating cancer.

While Adagrasib demonstrates remarkable efficacy as a sedative, it is not devoid of potential adverse effects.^[8,12]^ Understanding and analyzing such data is crucial in assessing the safety and efficacy of medications. To address this, a thorough investigation is conducted herein, utilizing data from the FDA Adverse Event (AE) Reporting System (FAERS) pertaining to Adagrasib. The safety of any medication is a paramount concern in healthcare, especially when considering its widespread use and potential impact on patient outcomes. Adverse events (AEs) associated with medications can vary widely, ranging from mild and transient effects to severe and life-threatening complications. Therefore, it is imperative to thoroughly evaluate the safety profile of Adagrasib to ensure its appropriate use in clinical practice. The utilization of FAERS data in this study offers a unique opportunity to gain insights into the real-world safety experience of patients receiving Adagrasib. FAERS serves as a valuable resource, providing a repository of AE reports submitted by healthcare professionals, patients, and manufacturers. By leveraging this data source, researchers can conduct signal detection analyses to identify potential safety concerns associated with Adagrasib use. Signal quantification methods, such as disproportionality analysis and Bayesian data mining algorithms, are employed to systematically evaluate the relationship between Adagrasib exposure and AEs. These methodologies allow for the detection of signals indicative of potential safety issues, providing valuable information for healthcare providers and regulatory agencies. Furthermore, the comprehensive nature of this analysis ensures that potential safety concerns associated with Adagrasib are thoroughly investigated and communicated to relevant stakeholders. By elucidating the safety profile of Adagrasib, healthcare providers can make informed decisions regarding its use in clinical practice, ultimately optimizing patient care and outcomes. In conclusion, this study intends to offer a thorough and meticulous analysis of Adagrasib safety profile by leveraging data from the FAERS and employing advanced signal quantification techniques. By integrating these sophisticated methods with a robust dataset, the research aims to yield precise and reliable insights into the adverse effects associated with Adagrasib. This comprehensive approach is expected to significantly contribute to the existing body of knowledge, ultimately aiding healthcare professionals in making more informed and safer treatment decisions.

## 2. Methods

### 2.1. Data source

This study extracted American Standard Code for Information Interchange report files from the FAERS database, spanning from the fourth quarter of 2022 to the first quarter of 2025, corresponding with the drug’s market introduction. The data was then imported into R for further analysis.

### 2.2. Data extraction and analysis

Redundant reports were removed, retaining only the latest report for each unique case ID based on the date in the demographics database. Data were linked via the “primaryid” field, with discrepancies in age and weight indicators rectified. Reports designating Adagrasib as the primary drug associated with analysis adverse drug event (ADEs) were chosen. These reports furnished details on the reporting date, patient’s age and gender, the reporter, and location. The study applied 4 disproportionality methods – reporting odds ratio (ROR),^[13]^ proportional reporting ratio (PRR),^[14]^Bayesian confidence propensity for neural networks,^[15]^ and MGPS^[16]^ – to detect drug ADE signals. ROR addresses bias st emming from limited reports on specific events, while PRR offers specificity. Bayesian confidence propensity for neural networks excels in integrating and cross-validating data from various sources, and MGPS can detect signals from rare events.

**Figure 1.**
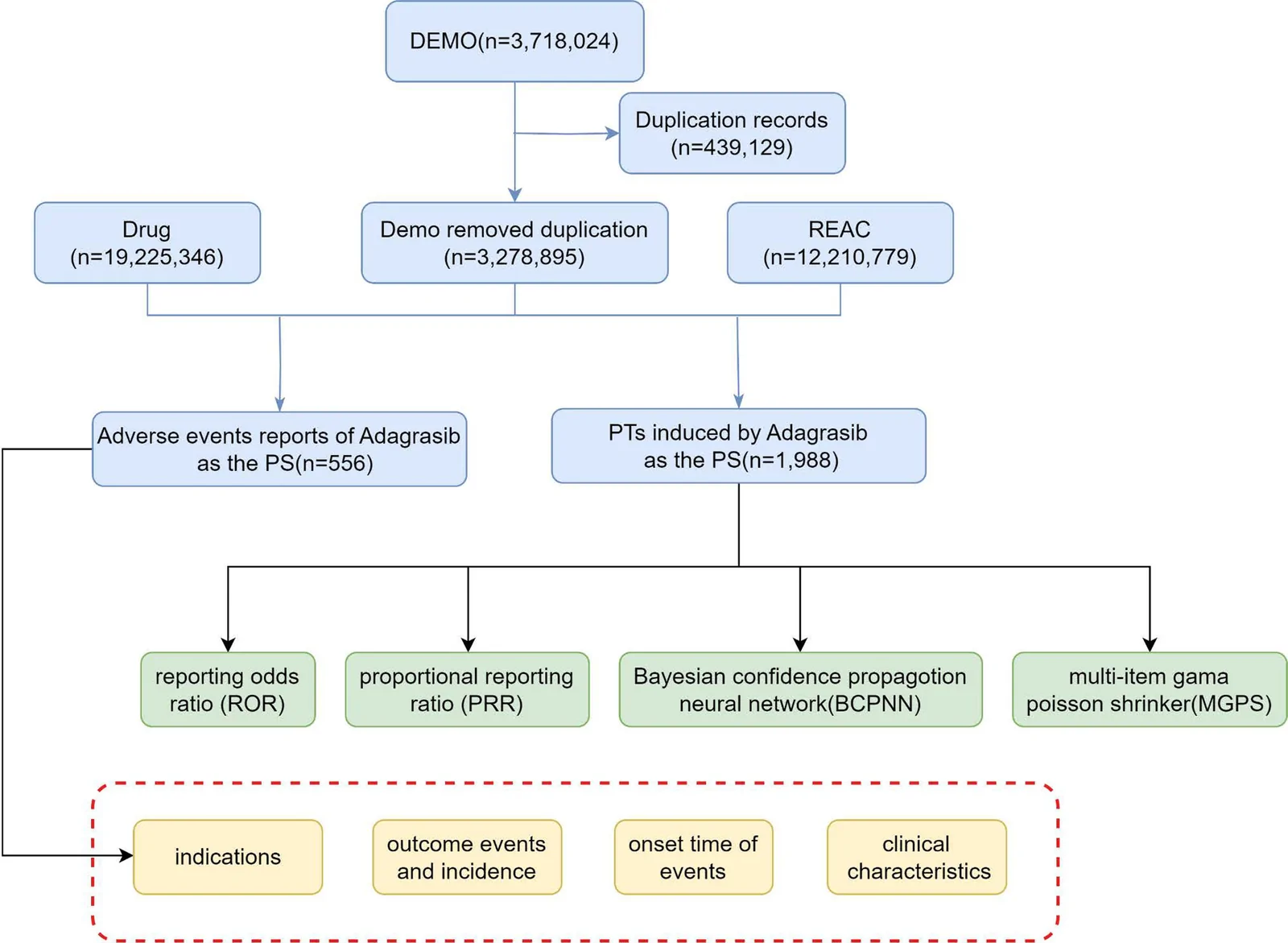
The flow diagram of selecting Adagrasib-related AEs from FAES database. AEs = adverse events.

The combined approach integrates the unique strengths of each algorithm, facilitating a comprehensive and nuanced analysis of AE data associated with Adagrasib. By leveraging multiple algorithms, this approach expands the detection scope, validates findings from diverse perspectives, and enhances the reliability of safety signal identification. Cross-validation across algorithms plays a pivotal role in mitigating false positives, ensuring that identified signals are robust and reproducible. Moreover, adjustments in thresholds and variance parameters enable the detection of rare adverse reactions, enhancing the sensitivity of signal detection algorithms. The utilization of 2 × 2 contingency tables, as outlined in Table 1, ensures a standardized framework for statistical analysis across methodologies. Precise calculations and threshold values, as delineated in Table 2, provide transparency and reproducibility in signal identification processes. Statistical analysis is conducted using Microsoft Excel 2022, offering a familiar and accessible platform for data manipulation and calculation. Subsequently, the data are imported into R software for further analysis, leveraging its advanced statistical capabilities and visualization tools. In this framework, higher values within the signal intensity metrics indicate a stronger association between Adagrasib exposure and AEs. This enables the prioritization of signals based on their significance and clinical relevance, facilitating informed decision-making regarding drug safety. Figure 1 visually elucidates the precise methodology employed for identifying AEs linked to Adagrasib within the FAERS database. Through a systematic and rigorous approach, this combined methodology aims to enhance our understanding of the safety profile of Adagrasib, ultimately informing clinical practice and regulatory decision-making processes.

**Table 1:** Four grid table.

	Adagrasib -related ADEs	Non- Adagrasib -related ADEs	Total
Adagrasib	*a*	*b*	*a + b*
Non- Adagrasib	*c*	*d*	*c + d*
Total	*a + c*	*b + d*	*N = a + b + c + d*

ADEs = analysis adverse drug events.

**Table 2:** Four main algorithms used to assess the potential association between Adagrasib and AEs.

Method	Formula	Threshold
ROR	$ROR=\frac{a/c}{b/d}$	*a* ≥3, ROR ≥3, 95% CI (lower limit) > 1
	$SE\left(lnROR\right)=\sqrt{\frac{1}{a}+\frac{1}{b}+\frac{1}{c}+\frac{1}{d}}$	
	$95\%\;CI=\;\;\;e^{\ln\left(ROR\right)\pm 1.96se}$	
PRR	$PRR=\frac{a/\left(a+b\right)}{c/\left(c+d\right)}$	*a* ≥3, PRR ≥2, 95% CI (lower limit) >1
	$SE\left(lnPRR\right)=\sqrt{\frac{1}{a}-\frac{1}{a+b}+\frac{1}{c}-\frac{1}{c+d}}$	
	$95\%\;CI=e^{\ln\left(PRR\right)\pm 1.96se}$	
BCPNN	$IC=log_{2}\frac{p\left(x,\;y\right)}{p\left(x\right)\;p\left(y\right)}=\;log_{2}\frac{a\left(a+b+c+d\right)}{\left(a+b\right)\left(a+c\right)}$	IC025 >0
	$E\left(IC\right)=log_{2}\frac{\left(a+\gamma 11\right)\left(a+b+c+d+\alpha \right)\left(a+b+c+d+\beta \right)}{\left(a+b+c+d+\gamma \right)\left(a+b+\alpha 1\right)\left(a+c+\beta 1\right)}$	
	$V\left(IC\right)=\frac{1}{{\left(ln2\right)}^{2}}\left[\frac{\left(a+b+c+d\right)-a+\gamma -\gamma 11}{\left(a+\gamma 11\right)\left(1+a+b+c+d+\gamma \right)}+\frac{\left(a+b+c+d\right)-\left(a+b\right)+a-\alpha 1}{\left(a+b+\alpha 1\right)\left(1+a+b+c+d+\alpha \right)}+\frac{\left(a+b+c+d+\alpha \right)-\left(a+c\right)+\beta -\beta 1}{\left(a+b+\beta 1\right)\left(1+a+b+c+d+\beta \right)}\right]$	
	$\gamma =\gamma 11\frac{\left(a+b+c+d+\alpha \right)\left(a+b+c+d+\beta \right)}{\left(a+b+\alpha 1\right)\left(a+c+\beta 1\right)}$	
	$IC-2SD=E\left(IC\right)-2\;\sqrt{V\left(IC\right)}$	
EBGM	$EBGM=\frac{a\left(a+b+c+d\right)}{\left(a+c\right)\left(a+b\right)}$	EBGM05 >2
	$SE\left(lnEBGM\right)=\sqrt{\frac{1}{a}+\frac{1}{b}+\frac{1}{c}+\frac{1}{d}}$	
	$95\%\;CI=\;\;\;e^{\ln\left(EBGM\right)\pm 1.96se}$	

AEs = adverse events, BCPNN = Bayesian Confidence Propagation Neural Network, PRR = proportional reporting ratios, ROR = reporting odds ratios.

### 2.3. Signal filtering and categorization

In the initial screening phase of this study, a stringent criterion was applied, filtering for preferred terms (PT) with reported counts of 3 or more. This approach aimed to prioritize AEs with a higher frequency of occurrence, ensuring the inclusion of signals that are more likely to be clinically relevant. The utilization of the Medical Dictionary for Regulatory Activities PT and System Organ Class (SOC) provided a standardized framework for encoding, categorizing, and identifying AE signals for analysis. By employing standardized terminology and classification systems, such as MedDRA, this study facilitated the systematic analysis of AE data, enabling researchers to identify patterns and trends across different organ systems and medical conditions. Specifically, the focus was directed towards the SOC associated with AE signals, allowing for a targeted investigation into specific physiological or anatomical systems affected by Adagrasib. This approach not only enhances the efficiency of signal detection but also provides valuable insights into the potential safety profile of Adagrasib across different patient populations and therapeutic indications. By focusing on AEs that meet predefined frequency thresholds and are categorized within specific organ classes, researchers can prioritize signals for further analysis and evaluation. Overall, the systematic screening and classification of AE data using standardized terminologies and classification systems lay the groundwork for a comprehensive analysis of the safety profile of Adagrasib. Through this approach, researchers aim to identify and characterize potential safety concerns associated with Adagrasib use, ultimately informing clinical decision-making and regulatory assessment processes.

## 3. Results

### 3.1. Basic characteristics of Adagrasib-related AE reports

**Figure 2.**
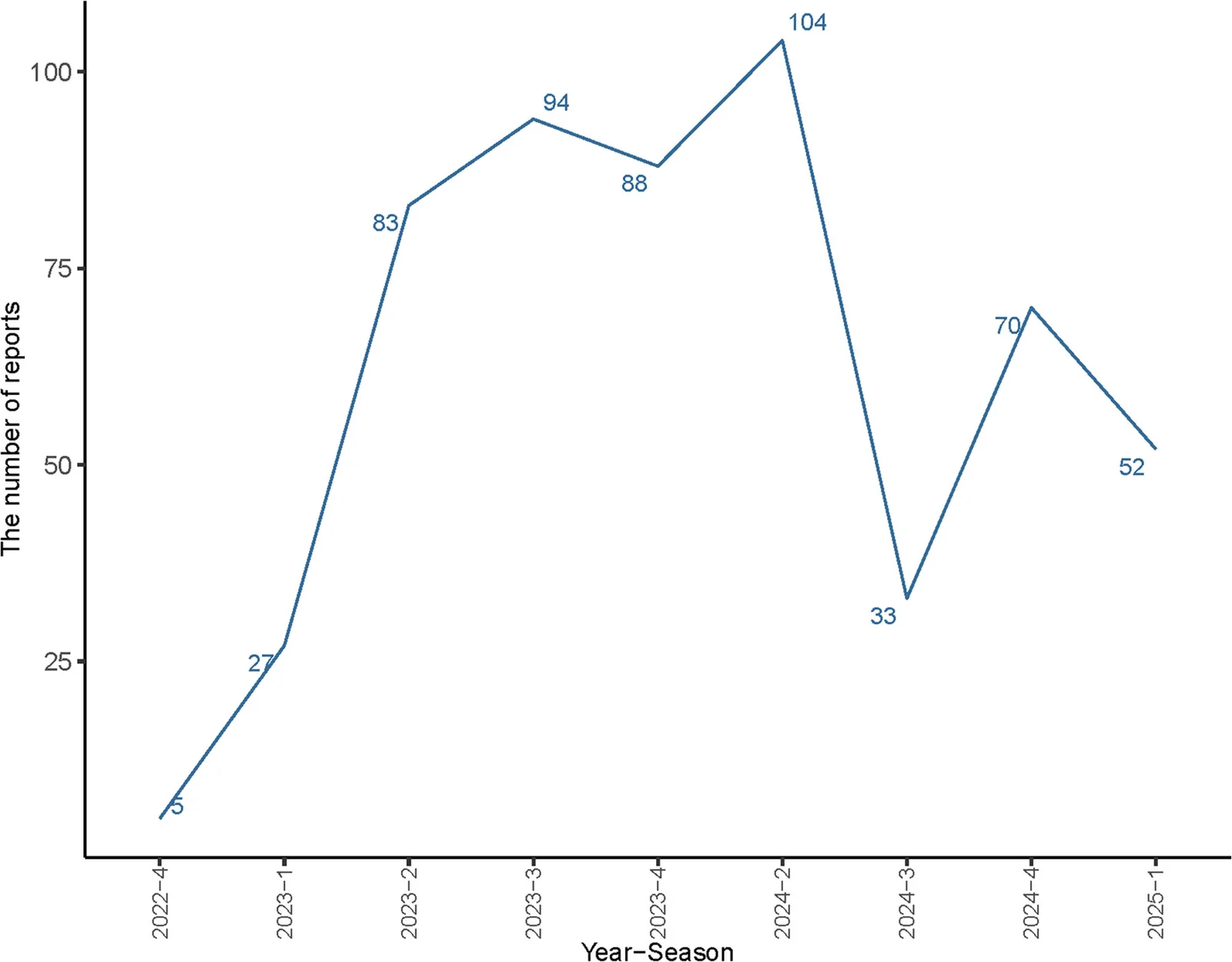
The number of AEs was reported quarterly after Adagrasib marketing.

The study extracted a total of 3718,024 AE reports from the FAERS database, covering the period from the fourth quarter of 2022 to the first quarter of 2025. Within this extensive dataset, Adagrasib was identified as the primary suspect in 556 ADE reports, as illustrated in Figure 2. This focused analysis of Adagrasib-related reports within the broader context of over one and a half million AE reports provides a substantial foundation for evaluating the safety profile of the drug. By isolating and examining these specific reports, the research aims to uncover detailed insights and patterns related to the adverse effects associated with Adagrasib use, thereby contributing to a more nuanced understanding of its safety in clinical practice.

The AE reports were almost evenly distributed between women (35.79%) and men (30.04%). However, a major challenge in the analysis was the absence of age information in 73.02% of the reports, which significantly hindered a thorough examination of the relationship between age and AEs. This lack of critical demographic data limited the ability to perform a comprehensive age-specific analysis, potentially obscuring important age-related trends and patterns in adverse reactions to Adagrasib. Consequently, the research’s ability to provide detailed insights into how different age groups might be affected by the drug was constrained, highlighting the need for more complete and accurate reporting in future pharmacovigilance efforts.

**Figure 3.**
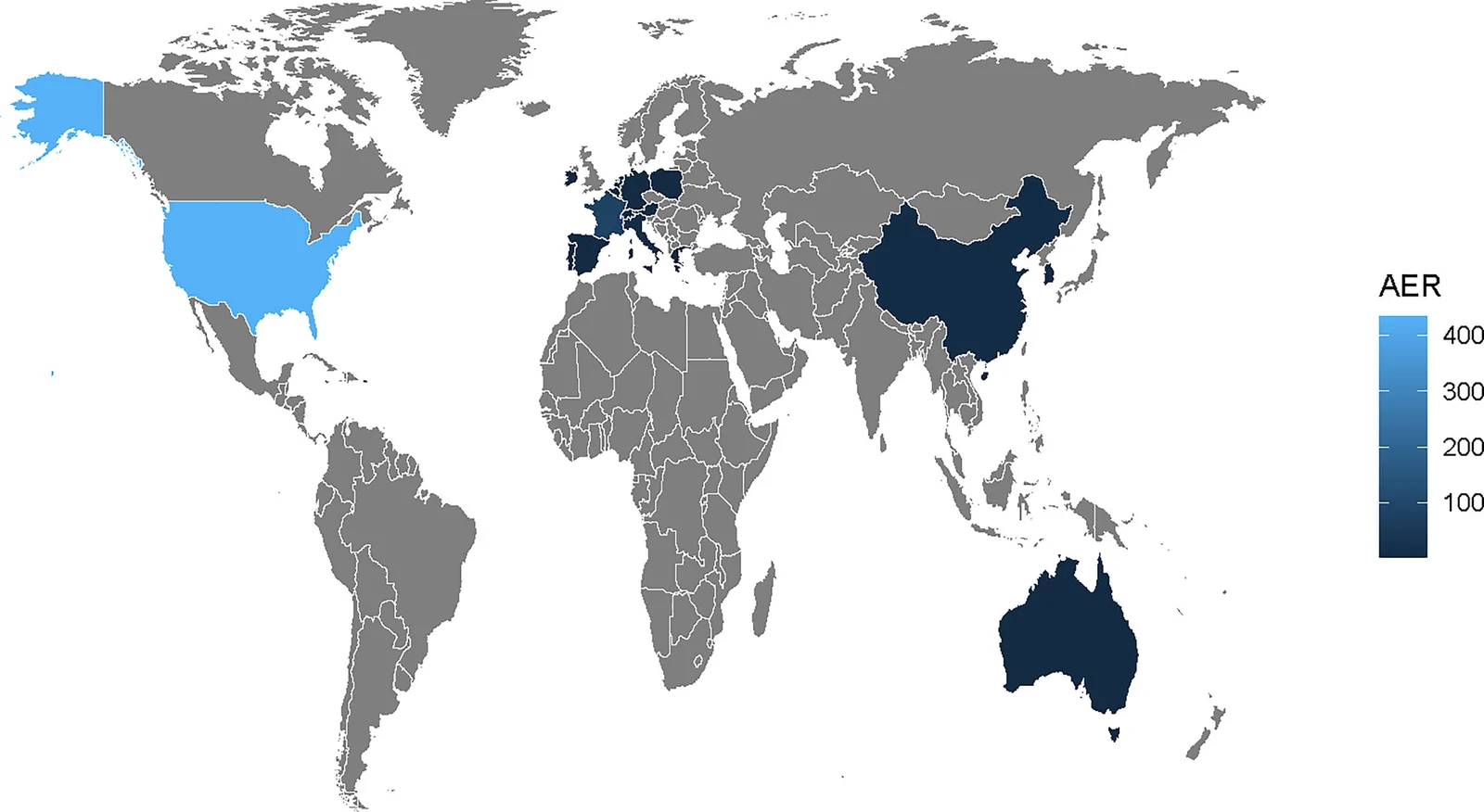
Distribution of the number of adverse reactions in the world.

The primary sources of reports were Consumers (48.92%), Pharmacists (27.52%), and Physicians (23.56%), with the majority (78.06%) originating from the US (Fig. 3). Regarding clinical outcomes, besides unspecified serious AEs, those leading to death were most common (36.66%), followed by hospitalization (31.93%). Further details are presented in Table 3.

**Table 3:** Characteristics of reports associated with Adagrasib from 2022 Q4–2025 Q1.

Variable	Number of events (%)
Year	
2022	5 (0.90)
2023	292 (52.52)
2024	207 (37.23)
2025	52 (9.35)
Sex	
Female	199 (35.79)
Male	167 (30.04)
Unknown	190 (34.17)
Age	
<18	0 (0.00)
18–45	3 (0.54)
45–65	42 (7.55)
65–75	61 (10.97)
≥75	44 (7.91)
Unknown	406 (73.02)
Reporter	
Consumer	272 (48.92)
Pharmacist	153 (27.52)
Physician	131 (23.56)
Reported countries	
United States	434 (78.06)
Other	122 (21.94)
Route	
Oral	435 (78.24)
Other	121 (21.76)
Outcomes	
Death	217 (36.66)
Hospitalization	189 (31.93)
Other serious	157 (26.52)
Life threatening	18 (3.04)
Disability	10 (1.69)
Required intervention to Prevent Permanent Impairment/Damage	1 (0.17)
Adverse event occurrence time – medication date (days)	
<7	59 (14.71)
7–28	95 (23.69)
28–60	50 (12.47)
≥60	63 (15.71)
Unknown	134 (33.42)

### 3.2. Adagrasib signal mining

#### 3.2.1. *Signal of system organ class*

**Figure 4.**
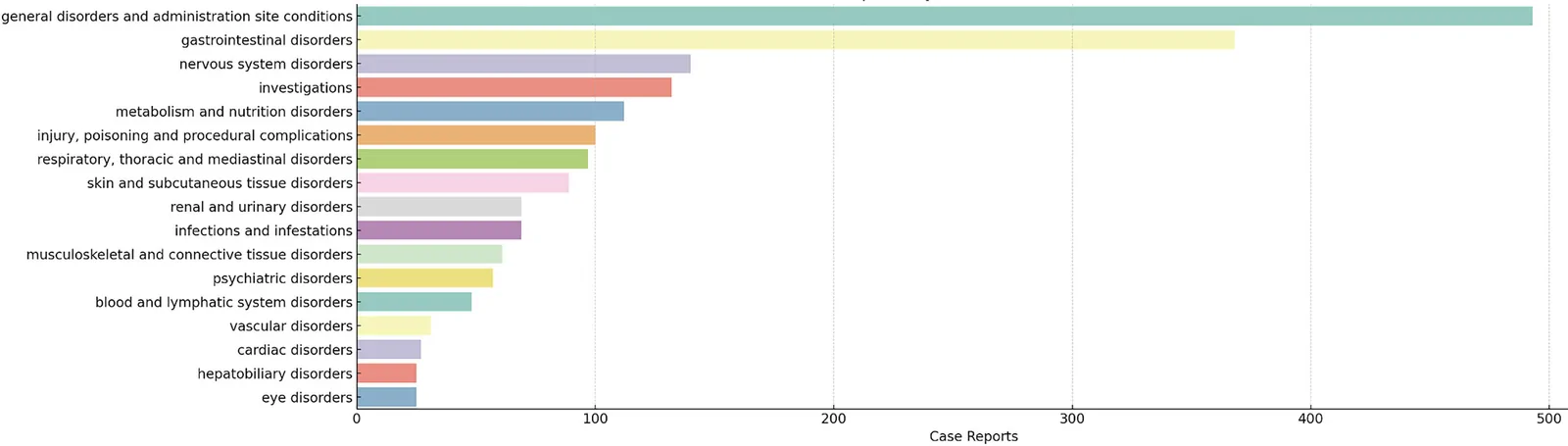
Adagrasib-related case distribution across SOCs. ADE = analysis adverse drug event, SOCs = system organ classifications.

Signal strengths of reports of Adagrasib at the SOC level are described in Figure 4. The study revealed that the 3 most frequent systems were general disorders and administration site conditions (n = 493, ROR 1.46, PRR 1.34, IC 0.43, EBGM 1.34), gastrointestinal disorders (n = 368 ROR 2.45, PRR 2.18, IC 1.12, EBGM 2.18) and nervous system disorders (n = 140, ROR 0.93, PRR 0.94, IC −0.09, EBGM 0.94), consistent with the characteristics of Adagrasib as an antitumor drug. Further details can be found in Table 4.

**Table 4:** Signal strength of reports of Adagrasib at the SOC level in FAERS database.

SOC	Case Reports	ROR (95% CI)	PRR (95% CI)	chisq	IC (IC025)	EBGM (EBGM05)
General disorders and administration site conditions	493	1.46 (1.32–1.61)	1.34 (1.24–1.45)	53.46	0.43 (0.29)	1.34 (1.24)
Gastrointestinal disorders	368	2.45 (2.18–2.74)	2.18 (1.98–2.4)	256.06	1.12 (0.96)	2.18 (1.98)
Nervous system disorders	140	0.93 (0.78–1.11)	0.94 (0.8–1.1)	0.65	−0.09 (−0.34)	0.94 (0.81)
Investigations	132	1.1 (0.92–1.31)	1.09 (0.93–1.28)	1.05	0.12 (−0.13)	1.09 (0.94)
Metabolism and nutrition disorders	112	2.85 (2.35–3.45)	2.74 (2.3–3.27)	126.72	1.46 (1.18)	2.74 (2.34)
Injury, poisoning and procedural complications	100	0.31 (0.26–0.38)	0.35 (0.29–0.43)	141.96	−1.52 (−1.81)	0.35 (0.29)
Respiratory, thoracic and mediastinal disorders	97	1.01 (0.82–1.24)	1.01 (0.83–1.23)	0.01	0.01 (−0.28)	1.01 (0.85)
skin and subcutaneous tissue disorders	89	0.76 (0.62–0.95)	0.78 (0.64–0.95)	6.14	−0.37 (−0.67)	0.78 (0.65)
Renal and urinary disorders	69	2.18 (1.71–2.77)	2.14 (1.69–2.71)	42.44	1.1 (0.75)	2.14 (1.75)
Infections and infestations	69	0.52 (0.41–0.66)	0.54 (0.43–0.68)	29.76	−0.9 (−1.25)	0.54 (0.44)
Musculoskeletal and connective tissue disorders	61	0.55 (0.42–0.71)	0.56 (0.43–0.72)	22.11	−0.83 (−1.2)	0.56 (0.45)
Psychiatric disorders	57	0.58 (0.44–0.75)	0.59 (0.46–0.76)	17	−0.76 (−1.14)	0.59 (0.47)
Blood and lymphatic system disorders	48	1.32 (0.99–1.76)	1.31 (1–1.72)	3.66	0.39 (−0.02)	1.31 (1.03)
Neoplasms benign, malignant and unspecified (incl cysts and polyps)	32	0.8 (0.56–1.13)	0.8 (0.56–1.14)	1.63	−0.32 (−0.82)	0.8 (0.6)
Vascular disorders	31	0.81 (0.57–1.16)	0.81 (0.57–1.15)	1.34	−0.3 (−0.8)	0.81 (0.61)
Cardiac disorders	27	0.7 (0.48–1.02)	0.7 (0.48–1.02)	3.56	−0.52 (−1.05)	0.7 (0.51)
Hepatobiliary disorders	25	1.36 (0.91–2.01)	1.35 (0.91–2)	2.32	0.44 (−0.12)	1.35 (0.97)
Eye disorders	25	0.58 (0.39–0.86)	0.59 (0.4–0.87)	7.42	−0.77 (−1.33)	0.59 (0.42)
Ear and labyrinth disorders	6	0.7 (0.31–1.55)	0.7 (0.31–1.56)	0.8	−0.52 (−1.59)	0.7 (0.36)
Endocrine disorders	4	0.65 (0.24–1.73)	0.65 (0.24–1.73)	0.76	−0.62 (−1.89)	0.65 (0.29)

FAERS = Food and Drug Administration Adverse Event Reporting System, PRR = proportional reporting ratios, ROR = reporting odds ratios, SOCs = system organ classifications.

#### 3.2.2. *Signal of PTs*

**Figure 5.**
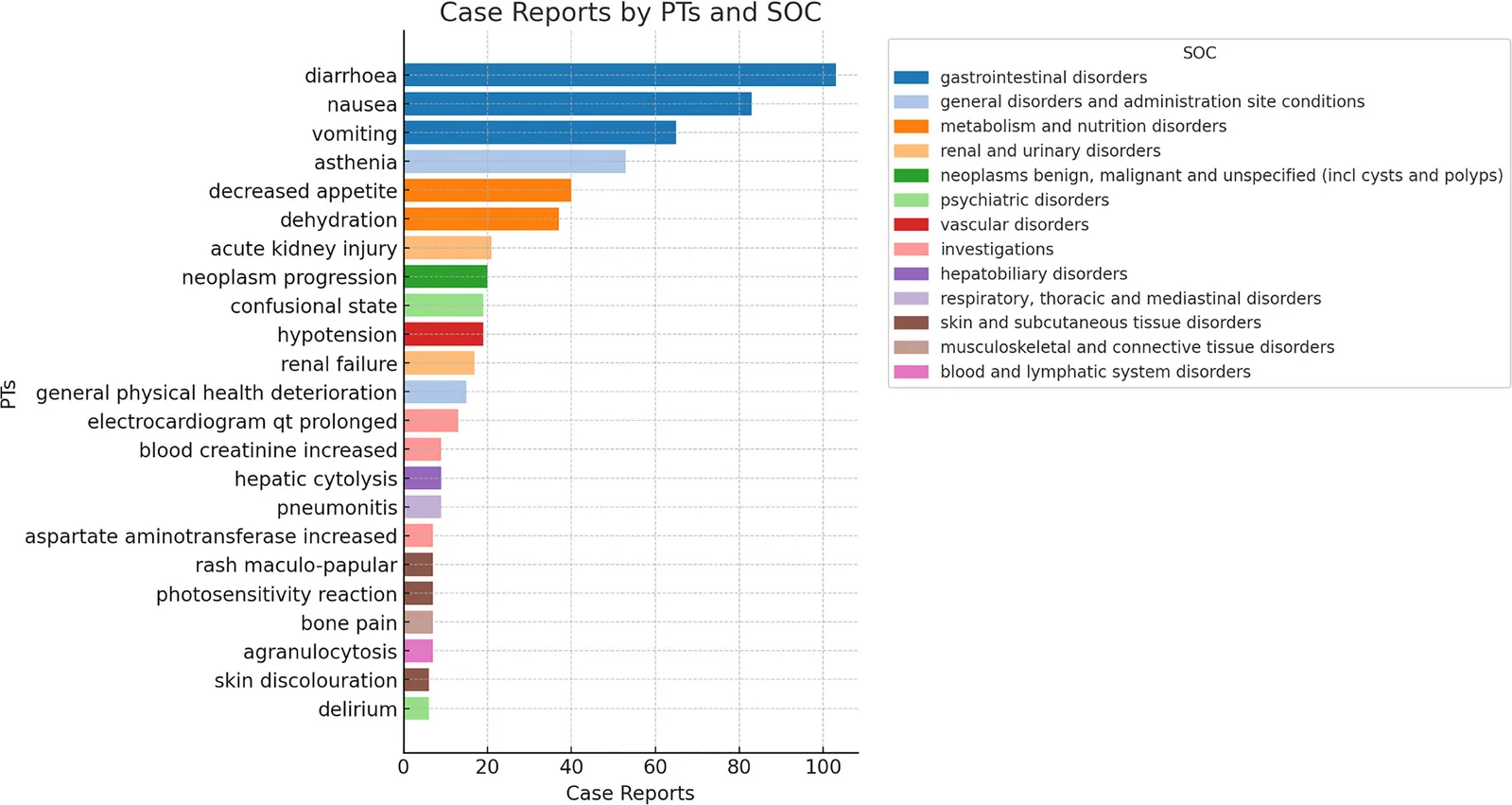
Bar graphs show the top 23AE signal intensities for Adagrasib in the FAERS database. The color represents the SOC corresponding to PT. The abscissa represents the number of reports. AEs = adverse events, FAERS = Food and Drug Administration Adverse Event Reporting System, PT = preferred terms, SOCs = system organ classifications.

At the PT level, this study used 4 algorithms to analyze adverse drug reactions and evaluate their compliance to various screening criteria, resulting in 38 PTs. According to the number of reports, the top 23 PTs are shown in Figure 5. The study showed that the 3 most PTs were death (n = 244), diarrhea (n = 103) and nausea (n = 83), details can be found in (Table S1, Supplemental Digital Content, https://links.lww.com/MD/Q78). It is worth noting that this study found some new, unlabeled adverse reactions, such as skin hyperpigmentation and seizure (Table S1, Supplemental Digital Content, https://links.lww.com/MD/Q78).

### 3.3. Onset time of events

**Figure 6.**
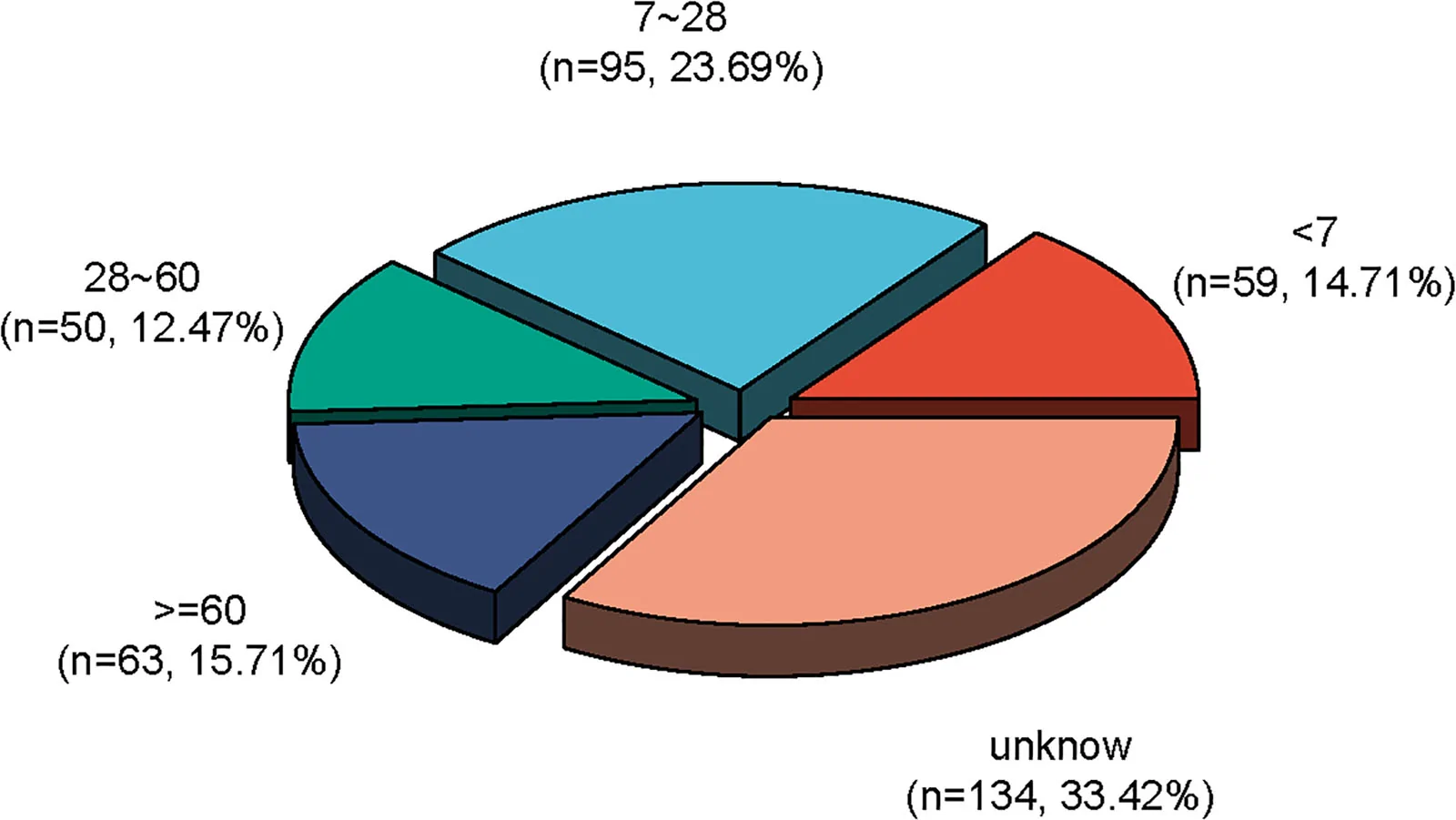
Time to onset of Adagrasib-related adverse AEs. AEs = adverse events.

59 AE cases (14.71%) occurred within 7 days after drug administration, while 95 AE cases (23.69%) occurred within 7 to 28 days after drug administration. 50 cases (12.47%) occurred within 28 to 60 days after drug administration, and 63 cases (15.71%) occurred after 60 days of drug administration. It is noteworthy that 33.42% of all adverse reaction reports lacked data regarding the time of occurrence. Further details can be found in Figure 6.

### 3.4. Gender-based difference in risk signals for Adagrasib

**Figure 7.**
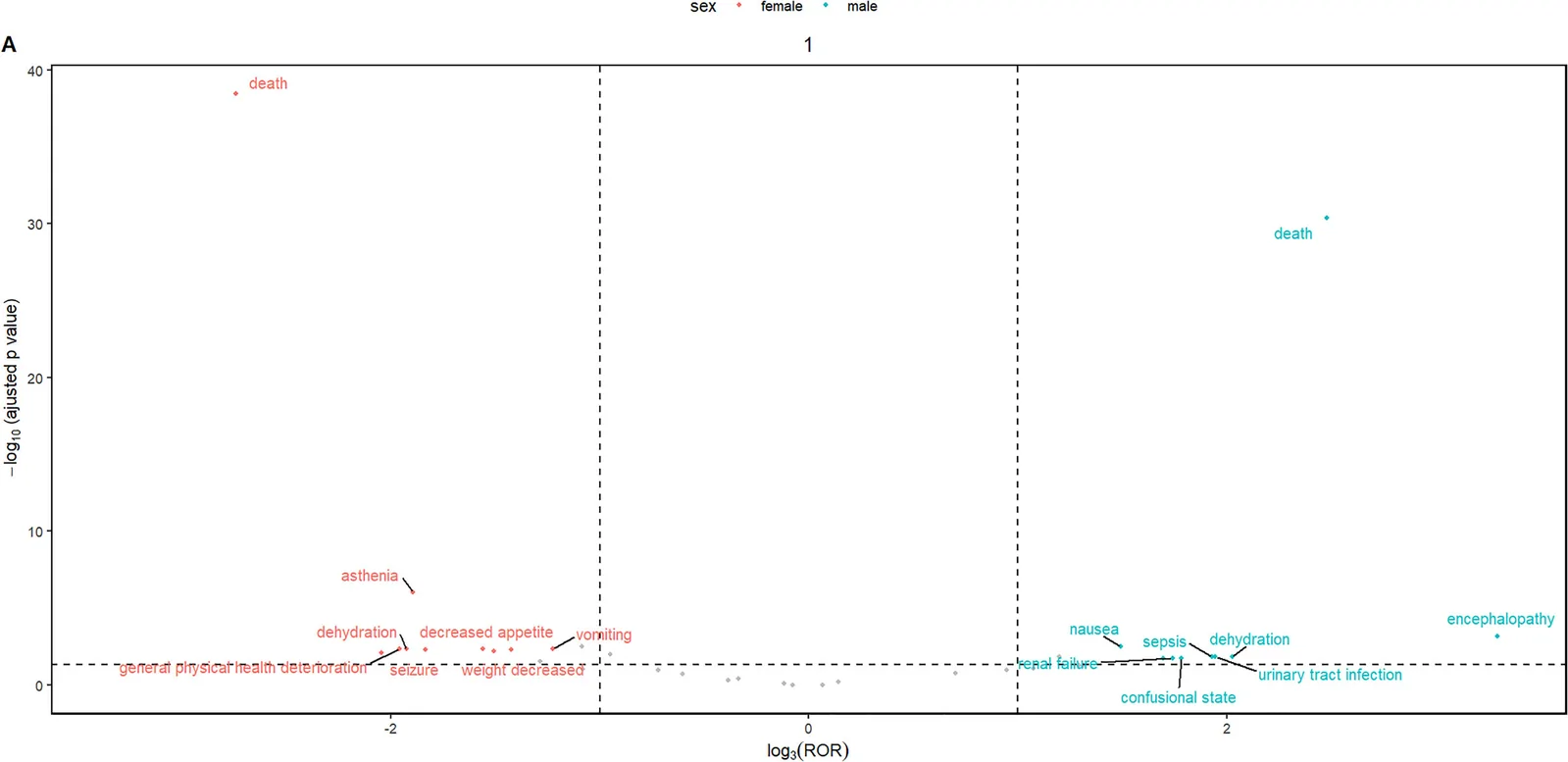
Gender-based difference in risk signals for Adagrasib.

In this study, a visual approach was adopted to conduct a comprehensive analysis of gender-based differences in ADE signals associated with Adagrasib, as illustrated in Figure 7. Each data point on the plot represents a specific ADE linked to Adagrasib, with particular emphasis placed on statistically significant ADEs. Notably, the visualization highlights distinct ADEs that exhibit statistical significance exclusively among either women or men. Among the ADEs significant only for women, notable manifestations include asthenia, general physical health deterioration, decreased appetite, seizure, weight loss, and vomiting. Conversely, ADEs unique to men encompass nausea, sepsis, renal failure, encephalopathy, confusion, dehydration and urinary tract infections. This visual representation offers a clear and direct comparison of how ADEs associated with Adagrasib manifest differently across genders. By elucidating gender-specific patterns in ADEs, this visualization underscores the importance of tailored approaches in clinical and therapeutic strategies. Recognizing and addressing gender-based differences in ADEs can inform personalized treatment decisions, optimize patient care, and minimize adverse outcomes. Furthermore, this analysis sheds light on potential underlying mechanisms or susceptibilities that may contribute to gender disparities in ADEs associated with Adagrasib. Such insights hold implications for drug development, regulatory evaluation, and clinical practice, emphasizing the need for gender-sensitive pharmacovigilance and risk management strategies. Overall, the visual analysis of gender-based differences in ADE signals associated with Adagrasib serves as a valuable tool for enhancing our understanding of drug safety and efficacy across diverse patient populations. By integrating gender-specific considerations into clinical decision-making processes, healthcare providers can optimize therapeutic outcomes and promote equitable healthcare delivery.

## 4. Discussion

The post-market surveillance of medication, especially regarding real-world usage and AEs, plays a pivotal role in ensuring ongoing safety and efficacy.^[17]^ We identified 130 positive AE signals related to Adagrasib, involving several systems, such as general disorders and administration site conditions, gastrointestinal disorders, infections and infestations, and nervous system disorders. The AEs associated with Adagrasib that were observed in previous clinical studies are consistent with our findings (Table 4 and Table S1, Supplemental Digital Content, https://links.lww.com/MD/Q78) and include diarrhea, nausea, vomiting, asthenia, and decreased appetite.^[8,11,18]^

Our comprehensive analysis has revealed several unexpected and previously unreported significant AE signals associated with Adagrasib administration, including skin hyperpigmentation and seizures. These findings underscore the importance of heightened vigilance among healthcare professionals regarding these severe and unforeseen AEs. The emergence of these AEs highlights risks that were not adequately captured during earlier regulatory trials.

The incidence of AEs associated with Adagrasib seems to exhibit comparability between female and male patients based on the available data. However, a notable limitation arises from a significant portion of the dataset lacking specific age details, which hinders a comprehensive understanding of AE frequencies across different age groups. Future research endeavors should prioritize obtaining precise age information and delving into potential variations in drug reactions among diverse age demographics. Moreover, it is essential to highlight that only a minority of cases, accounting for 23.56%, were reported by physicians, indicating a substantial gap in physician reporting within the FAERS database. This raises concerns about underreporting and underscores the importance of enhancing healthcare provider awareness and engagement in pharmacovigilance activities. Increased physician reporting can enrich the quality and comprehensiveness of AE data, thereby facilitating more accurate risk assessments and informed decision-making in clinical practice. Furthermore, the overwhelming majority of AE reports originated from the United States, comprising 78.06% of the dataset. This observation suggests potential regional or cultural patterns in reporting practices, which warrant further investigation. Understanding any regional or cultural biases in AE reporting is crucial for interpreting and generalizing findings from pharmacovigilance studies conducted using FAERS data. In conclusion, while the current analysis provides valuable insights into the incidence of AEs associated with Adagrasib, several important considerations and limitations emerge. Addressing these limitations, such as the lack of specific age details and the gap in physician reporting, is essential for advancing pharmacovigilance efforts and enhancing the safety monitoring of Adagrasib and other medications. Additionally, exploring potential regional or cultural biases in AE reporting can contribute to a more comprehensive understanding of drug safety profiles and improve public health outcomes.

In this study, we conducted a comprehensive and systematic analysis of the FAERS database to evaluate the postmarketing safety characteristics of Adagrasib. However, it is important to acknowledge that FAERS is a spontaneous reporting system with variability in the completeness and accuracy of information, which can introduce biases in data analysis. Underreporting is a significant issue due to the voluntary nature of the reports, and the reports in the database do not establish causality, only indicating potential adverse AEs. Additionally, several unmeasured confounding factors, such as potential drug interactions, comorbidities, and concurrent medications, were not included in the study, adding complexity to the interpretation of the data. Despite these limitations, our research identified 2 unexpected and significant AEs, including disorientation and seizure, thus expanding the safety information known from clinical trial phases. These findings provide valuable references for healthcare professionals to closely follow up with patients and monitor drug-related AEs in clinical practice. We recognize that while FAERS data provide preliminary signals about potential risks, determining the true incidence and causality requires further validation through prospective clinical trials. This study not only supplements the information in the FAERS rare AE system but also provides direction for future research, advancing our understanding of the safety of Adagrasib in real-world clinical settings.

## 5. Conclusion

In conclusion, the present study analyzed Adagrasib real-world AEs using the FAERS database from 2022 Q4 to 2025 Q1. The study provided a comprehensive safety profile, identifying known risks and uncovering new signals, such as skin hyperpigmentation and seizures. The findings emphasize the necessity for continuous monitoring, enhanced reporting practices, and additional investigation into biases. Despite the limitations of FAERS data, the present study offers valuable insights for clinical decision-making and patient care. Further validation through clinical trials is required to confirm incidence and causality. This research contributes to the advancement of knowledge regarding the safety of Adagrasib, thereby informing future studies and medical practice.

## Author contributions

**Data curation:** Sijing Tu.

**Formal analysis:** Sidi Jian, Sheng Chen, Xuan Lan, Peiling Zuo.

**Investigation:** Sidi Jian, Shuang Liang, Encun Hou, Sijing Tu.

**Methodology:** Sidi Jian, Shuang Liang, Xiaohan Ma, Sheng Chen, Xiaofei Zhang, Xuan Lan, Peiling Zuo, Encun Hou, Sijing Tu.

**Resources:** Sidi Jian.

**Software:** Sidi Jian, Shuang Liang, Xiaohan Ma, Sheng Chen, Xiaofei Zhang, Xuan Lan, Peiling Zuo, Sijing Tu.

**Supervision:** Shuang Liang, Sijing Tu.

**Validation:** Shuang Liang, Sijing Tu.

**Visualization:** Shuang Liang, Sijing Tu.

**Writing – original draft:** Sidi Jian, Shuang Liang, Xiaohan Ma, Peiling Zuo, Sijing Tu.

**Writing – review & editing:** Sidi Jian, Shuang Liang, Xiaohan Ma, Xiaofei Zhang, Sijing Tu.

## Supplementary Material
