# Supplementary material for: Real-world adverse event analysis of Adagrasib: Insights from the FAERS database
Source: Medicine (Baltimore). 2025 Sep 26;104(39):e44755. doi: 10.1097/MD.0000000000044755 (PMC13593117; doi:10.1097/MD.0000000000044755)
Supplement: Supplementary file 1 [file medi-104-e44755-s001.docx]

Table S1. All PT reports for Adagrasib.

| SOC | PT | Case Reports | ROR (95% CI) | PRR (95% CI) | chisq | IC (IC025) | EBGM (EBGM05) |
| --- | --- | --- | --- | --- | --- | --- | --- |
| investigations | electrocardiogram qt prolonged | 13 | 11.67(6.76, 20.15) | 11.6(6.7, 20.08) | 125.76 | 3.53(2.77) | 11.58(7.33) |
| investigations | blood creatinine increased | 9 | 4.88(2.53, 9.4) | 4.86(2.55, 9.28) | 27.62 | 2.28(1.38) | 4.86(2.81) |
| investigations | aspartate aminotransferase increased | 7 | 5.24(2.5, 11.02) | 5.23(2.48, 11.01) | 23.93 | 2.39(1.38) | 5.22(2.81) |
| investigations | gamma-glutamyltransferase increased | 4 | 8.42(3.15, 22.47) | 8.4(3.15, 22.38) | 26.06 | 3.07(1.8) | 8.39(3.69) |
| investigations | blood sodium decreased | 4 | 7.63(2.86, 20.36) | 7.61(2.86, 20.28) | 22.96 | 2.93(1.66) | 7.61(3.35) |
| investigations | international normalised ratio increased | 3 | 11.26(3.62, 34.97) | 11.24(3.61, 35.03) | 27.94 | 3.49(2.07) | 11.22(4.35) |
| investigations | blood magnesium decreased | 3 | 9.08(2.92, 28.21) | 9.07(2.91, 28.27) | 21.52 | 3.18(1.76) | 9.06(3.51) |
| investigations | blood potassium increased | 3 | 7.28(2.34, 22.6) | 7.27(2.33, 22.66) | 16.2 | 2.86(1.44) | 7.26(2.81) |
| skin and subcutaneous tissue disorders | rash maculo-papular | 7 | 9.82(4.67, 20.65) | 9.79(4.65, 20.62) | 55.19 | 3.29(2.29) | 9.78(5.25) |
| skin and subcutaneous tissue disorders | photosensitivity reaction | 7 | 14.36(6.83, 30.19) | 14.32(6.8, 30.16) | 86.52 | 3.84(2.83) | 14.28(7.67) |
| skin and subcutaneous tissue disorders | skin discolouration | 6 | 4.63(2.08, 10.33) | 4.62(2.07, 10.32) | 17.04 | 2.21(1.14) | 4.62(2.36) |
| skin and subcutaneous tissue disorders | skin hyperpigmentation | 4 | 20.89(7.82, 55.82) | 20.85(7.83, 55.55) | 75.36 | 4.38(3.11) | 20.79(9.13) |
| skin and subcutaneous tissue disorders | toxic skin eruption | 3 | 10.42(3.36, 32.38) | 10.41(3.34, 32.45) | 25.48 | 3.38(1.96) | 10.39(4.03) |
| gastrointestinal disorders | diarrhoea | 103 | 4.81(3.94, 5.86) | 4.61(3.79, 5.61) | 294.41 | 2.2(1.92) | 4.61(3.9) |
| gastrointestinal disorders | nausea | 83 | 3.64(2.92, 4.53) | 3.53(2.85, 4.38) | 151.88 | 1.82(1.5) | 3.52(2.93) |
| gastrointestinal disorders | vomiting | 65 | 4.77(3.72, 6.11) | 4.65(3.68, 5.88) | 187.13 | 2.22(1.86) | 4.64(3.78) |
| gastrointestinal disorders | retching | 5 | 7.99(3.32, 19.22) | 7.97(3.3, 19.25) | 30.44 | 2.99(1.84) | 7.96(3.82) |
| psychiatric disorders | confusional state | 19 | 4.39(2.79, 6.9) | 4.36(2.78, 6.84) | 49.19 | 2.12(1.49) | 4.35(2.98) |
| psychiatric disorders | delirium | 6 | 5.24(2.35, 11.69) | 5.23(2.34, 11.68) | 20.52 | 2.39(1.31) | 5.23(2.67) |
| psychiatric disorders | mental status changes | 5 | 8.78(3.65, 21.13) | 8.76(3.63, 21.16) | 34.32 | 3.13(1.97) | 8.75(4.19) |
| metabolism and nutrition disorders | decreased appetite | 40 | 5(3.66, 6.84) | 4.92(3.6, 6.73) | 125.46 | 2.3(1.85) | 4.92(3.79) |
| metabolism and nutrition disorders | dehydration | 37 | 10.82(7.82, 14.99) | 10.64(7.78, 14.56) | 323.18 | 3.41(2.95) | 10.62(8.09) |
| metabolism and nutrition disorders | feeding disorder | 4 | 4.57(1.71, 12.18) | 4.56(1.71, 12.15) | 11.11 | 2.19(0.92) | 4.56(2) |
| general disorders and administration site conditions | death | 244 | 10.36(9.06, 11.84) | 9.21(8.19, 10.36) | 1806.43 | 3.2(3.01) | 9.19(8.22) |
| general disorders and administration site conditions | asthenia | 53 | 4.6(3.5, 6.05) | 4.51(3.43, 5.93) | 145.48 | 2.17(1.78) | 4.51(3.59) |
| general disorders and administration site conditions | general physical health deterioration | 15 | 3.26(1.96, 5.42) | 3.24(1.95, 5.39) | 23.29 | 1.7(0.99) | 3.24(2.12) |
| renal and urinary disorders | acute kidney injury | 21 | 3.8(2.47, 5.85) | 3.77(2.45, 5.8) | 42.9 | 1.92(1.31) | 3.77(2.63) |
| renal and urinary disorders | renal failure | 17 | 6.01(3.73, 9.69) | 5.97(3.73, 9.56) | 70.31 | 2.58(1.91) | 5.96(4) |
| nervous system disorders | status epilepticus | 5 | 16.79(6.97, 40.44) | 16.75(6.93, 40.46) | 73.87 | 4.06(2.9) | 16.71(8.01) |
| nervous system disorders | encephalopathy | 4 | 5.14(1.92, 13.7) | 5.13(1.93, 13.67) | 13.28 | 2.36(1.09) | 5.12(2.25) |
| hepatobiliary disorders | hepatic cytolysis | 9 | 7.99(4.15, 15.38) | 7.96(4.17, 15.2) | 54.7 | 2.99(2.09) | 7.95(4.59) |
| hepatobiliary disorders | hepatotoxicity | 4 | 5.23(1.96, 13.96) | 5.22(1.96, 13.91) | 13.65 | 2.38(1.12) | 5.22(2.3) |
| vascular disorders | hypotension | 19 | 3.3(2.1, 5.18) | 3.28(2.09, 5.15) | 30.11 | 1.71(1.08) | 3.27(2.24) |
| respiratory, thoracic and mediastinal disorders | pneumonitis | 9 | 8.68(4.51, 16.72) | 8.65(4.53, 16.52) | 60.82 | 3.11(2.21) | 8.64(4.99) |
| musculoskeletal and connective tissue disorders | bone pain | 7 | 4.16(1.98, 8.73) | 4.15(1.97, 8.74) | 16.71 | 2.05(1.05) | 4.14(2.23) |
| injury, poisoning and procedural complications | sunburn | 4 | 23.73(8.88, 63.42) | 23.69(8.89, 63.12) | 86.6 | 4.56(3.29) | 23.6(10.37) |
| cardiac disorders | pericardial effusion | 5 | 7.13(2.96, 17.16) | 7.11(2.94, 17.18) | 26.25 | 2.83(1.67) | 7.11(3.41) |
| blood and lymphatic system disorders | agranulocytosis | 7 | 10.05(4.78, 21.13) | 10.02(4.76, 21.1) | 56.79 | 3.32(2.32) | 10.01(5.38) |
|  |  |  |  |  |  |  |  |
